# Improving performance in the ED through laboratory information exchange systems

**DOI:** 10.1186/s12245-018-0179-6

**Published:** 2018-03-12

**Authors:** Louis Raymond, Guy Paré, Éric Maillet, Ana Ortiz de Guinea, Marie-Claude Trudel, Josianne Marsan

**Affiliations:** 1 Université du Québec à Trois-Rivières, Trois-Rivières, Canada; 2 HEC Montréal, 3000, Cote-Sainte-Catherine Road, Montreal, Quebec H3T 2A7 Canada; 3 Université de Sherbrooke, Sherbrooke, Canada; 4 Université Laval, Quebec City, Canada

**Keywords:** Laboratory information exchange, Electronic health record system, Emergency medicine, Information technology, Survey

## Abstract

**Background:**

The accessibility of laboratory test results is crucial to the performance of emergency departments and to the safety of patients. This study aims to develop a better understanding of which laboratory information exchange (LIE) systems emergency care physicians (ECPs) are using to consult their patients’ laboratory test results and which benefits they derive from such use.

**Methods:**

A survey of 163 (36%) ECPs in Quebec was conducted in collaboration with the Quebec’s Department of Health and Social Services. Descriptive statistics, chi-square tests, cluster analyses, and ANOVAs were conducted.

**Results:**

The great majority of respondents indicated that they use several LIE systems including interoperable electronic health record (iEHR) systems, laboratory results viewers (LRVs), and emergency department information systems (EDIS) to consult their patients’ laboratory results. Three distinct profiles of LIE users were observed. The extent of LIE usage was found to be primarily determined by the functional design differences between LIE systems available in the EDs. Our findings also indicate that the more widespread LIE usage, the higher the perceived benefits. More specifically, physicians who make extensive use of iEHR systems and LRVs obtain the widest range of benefits in terms of efficiency, quality, and safety of emergency care.

**Conclusions:**

Extensive use of LIE systems allows ECPs to better determine and monitor the health status of their patients, verify their diagnostic assumptions, and apply evidence-based practices in laboratory medicine. But for such benefits to be possible, ECPs must be provided with LIE systems that produce accurate, up-to-date, complete, and easy-to-interpret information.

## Background

The accessibility, turnaround time, reliability, and predictive ability of laboratory test results are crucial to the performance of emergency departments (EDs) in hospitals [1–4], and to the safety and quality of care provided by emergency physicians (EPs) [5, 6]. Due to the specific nature of emergency medicine, EPs must rapidly investigate the state and stability of a patient’s health in order to make informed decisions and implement medical interventions that can make the difference between life and death. To do so, they must both consult the patient’s prior laboratory test results, ordered by other primary care and/or hospital physicians, and order new tests if necessary. Many EPs now achieve this through a health information exchange (HIE) platform that uses information technology (IT) to enable the interoperability of clinical data [7, 8].

In the ED, various types of information systems such as electronic health record systems, clinical information systems, laboratory information systems, and/or emergency department information systems (EDIS) may be used by EPs for laboratory medicine purposes [9–12]. EPs may also access HIE platforms such as regional data repositories set up by governmental or private organizations to enable the fluid circulation of information between primary care medical clinics, hospitals, and private laboratories [13, 14]. As HIE usage in the ED can take different forms, the interoperability of these systems has become a critical condition for extensive and effective usage by EPs [15, 16].

The “laboratory” component of HIE, called laboratory information exchange (LIE), has become an essential aspect of the quest to improve the quality, safety, timeliness, and cost-effectiveness of medical care in general [17] and of emergency care in particular [18–20]. In this regard, various motivations for LIE use have been identified, including improving the ED’s workflow and EP’s clinical judgment, overcoming informational or technical problems with the hospital’s electronic medical record system, and EP detecting drug interactions and misuses [21–23]. Moreover, LIE can be used to reduce ED costs related to test duplication and repeated patient visits as well as to improve patient follow-ups due to a greater integration of care services [24, 25].

The benefits of providing timely and relevant laboratory information to EPs are not limited to improved emergency care quality, and they go beyond the ED by reducing the hospitalization rate, the use of hospital resources (e.g., less imaging/lab tests, fewer consultations), and the length of patients’ hospital stays after ED visits [26, 27]. However, LIE usage may vary greatly from one physician to another, implying major differences in the barriers faced by EPs in their adoption of LIE, in the nature and extensiveness of their use of LIE, in the benefits eventually obtained from such use, and, most importantly, in the impact of such use upon their clinical decision-making and the well-being of their patients [28–30].

Given the need to deepen our understanding of the nature and effectiveness of EPs’ use of LIE systems, this study seeks to answer the following research questions: What is the nature of LIE usage in the ED, and, in particular, what types of information systems are actually used by EPs for laboratory medicine purposes? How extensive is this use? What are the benefits obtained by EPs from their LIE usage?

## Methods

As part of a larger research program on the use of LIE systems by physicians in Quebec, Canada, this study was designed as a self-administered survey. As described below, the online survey was designed and reported according to best practices [31]. The questionnaire instrument was developed following a comprehensive review of the extant literature on LIE and a series of in-depth face-to-face interviews with eight practicing EPs. Survey respondents were recruited with the help of the Quebec’s Ministry of Health and Social Services, which sent an invitation letter via email to all physicians who had an authorized access to the province-wide interoperable electronic health record (iEHR) called “Dossier Santé Québec.” The letter contained a hyperlink and a QR code for mobile devices, directing respondents to a secure Web page giving them access to the survey. Developed with the Qualtrics online survey platform [32], the questionnaire instrument was first approved by the Quebec health authorities and then pre-tested with two EPs. Each participant was interviewed regarding the content, format, instructions, questions, and responses to ensure he or she interpreted the questions and responses as presented and intended. Following minor adjustments to the questionnaire, the study received final approval from the ethics board of each researcher’s university. Two reminder letters were sent to all intended respondents 1 and 2 weeks after the initial invitation sent in early June 2016. No research incentives were used.

Our sample is composed of 163 EPs, representing 36% of the 456 physicians who were practicing in EDs in Quebec at the time of the survey. The possibility of a response bias in the data was assessed by comparing the 37 “late” respondents (i.e., those who responded after receiving the second reminder) with the 126 “early” respondents. No significant differences were found between these two groups, thus minimizing the potential for such a bias. The data were then analyzed through descriptive statistics as well as chi-square analysis, cluster analysis, and ANOVA, using the SPSS statistical package. As there were very few missing data (three respondents omitting the size of their ED), these were replaced by the mean value. The internal validity of the (index) measures of LIE use was confirmed though “item analysis,” that is, by confirming that each measure correlated sufficiently with the individual items that compose it [33].^1^ The internal validity of the measures of the performance outcomes of LIE use was confirmed through Cronbach’s *α* coefficient (with values ranging from 0.62 to 0.86).

## Results

As shown in Table 1, 51% of the EPs in our sample were women. In terms of age, 39% were in their 50s, 29% in their 40s, and 18% in their 30s. In terms of clinical experience, 39% had less than 10 years of experience, 37% had 10 to 19 years, and 24% had 20 years or more. Comparing the 130 generalists to the 33 specialists (i.e., board certified in emergency medicine) indicates that the latter group had a higher percentage of men, and its members were older and had more clinical experience, on average, than the respondents in the former group.

**Table 1:** Profile of the sample

Characteristics of the EPs	All physicians (*n* = 163)	Generalists (*n* = 130)	Specialists (*n* = 33)	*χ*^2^ test
	*n* (%)	*n* (%)	*n* (%)	
Gender				
Female	83 (51)	74 (57)	9 (27)	8.1**
Male	80 (49)	56 (43)	24 (73)	
Age				
30 years or younger	15 (9)	15 (12)	0 (0)	16.6**
30–39 years	63 (39)	51 (39)	12 (36)	
40–49 years	48 (29)	42 (32)	6 (18)	
50–59 years	30 (18)	19 (15)	11 (33)	
60 years or older	7 (4)	3 (2)	4 (12)	
Clinical experience				
5 years or less	31 (19)	27 (21)	4 (12)	17.3**
5–9 years	33 (20)	26 (20)	7 (21)	
10–14 years	32 (20)	28 (22)	4 (12)	
15–19 years	27 (17)	24 (18)	3 (9)	
20–24 years	10 (6)	9 (7)	1 (3)	
25 years or more	30 (18)	16 (12)	14 (42)	

**The *χ*^2^ value indicates a significant difference (*p* < 0.01) between generalists and specialists

Table 2 indicates that 38% of the physicians in our sample practiced in small- or medium-size EDs (5 to 20 EPs), and 62% practiced in large ones (21 to 62 EPs). As to their location, 61% practiced in an ED located in a central or urban region, whereas 39% practiced in peripheral or rural regions. Unsurprisingly, the medical specialists were more likely to practice in larger EDs located in urban regions when compared with the generalists.

**Table 2:** Characteristics of the sampled EPs’ emergency departments

Characteristics of the EDs	All physicians (*n* = 163)	Generalists (*n* = 130)	Specialists (*n* = 33)	*χ*^2^ test
	*n* (%)	*n* (%)	*n* (%)	
Size of the ED				
5–10 EPs	16 (10)	16 (12)	0 (0)	18.5***
11–20 EPs	46 (28)	44 (34)	2 (6)	
21–30 EPs	71 (44)	49 (38)	22 (67)	
31–40 EPs	25 (15)	17 (13)	8 (24)	
41–62 EPs	5 (3)	4 (3)	1 (3)	
Location of the ED				
Central/urban region	100 (61)	70 (46)	30 (57)	13.7***
Peripheral/rural region	63 (39)	60 (54)	3 (43)	

***The *χ*^2^ value indicates a significant difference (*p* < 0.001) between generalists and specialists

**Fig. 1:**
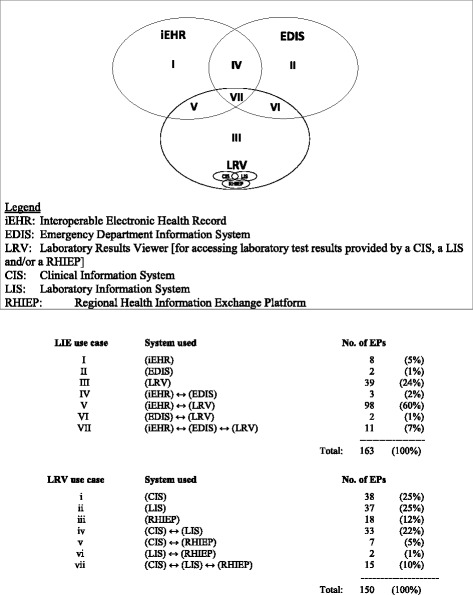
Nature of LIE use in the ED

As shown in Fig. 1, 120 of the EPs in our sample (74%) consult laboratory test results through the province-wide iEHR, only 18 (11%) through an EDIS, and 150 (92%) through a laboratory results “viewer” (LRV). In simple terms, an LRV is a common interface that allows physicians to access test results from their hospital’s electronic medical record, a public or private medical laboratory’s LIS, and/or their region’s HIE platform [34, 35]. In the case of EDIS, the great majority of EPs do not use such systems for laboratory medicine purposes mainly because they perceive these systems as having no particular LIE functionalities. A majority of EPs (70%) retrieve lab results through more than one information system, that is, through the iEHR in combination with an EDIS and/or an LRV. Moreover, the EPs may also use both an EDIS and an LRV (but not the iEHR) to order new lab tests. Conversely, only 30% of the EPs in our sample use a single source to retrieve lab test results: either the iEHR (4%), an EDIS (1%), or an LRV (24%).

Table 3 reveals important differences in the LIE functionalities available to the EPs in each type of system and in the actual use made of these functionalities by physicians. For instance, the ability to request a new lab test is available to 61% of the LRV users and to 39% of the EDIS users, whereas the ability to access regional lab results is available to 31% of LRV users. However, physicians seem to use most of the LIE functionalities available to them, utilizing on average 97, 83, and 86% of the functionalities available in the iEHR, their LRV, and their EDIS, respectively. A notable exception is that only 27% of LRV users request new lab tests through their system, even though this functionality is available in 61% of these systems.

**Table 3:** LIE functionalities as used by EPs

LIE capability	Availability of functionality (% of systems)	Use of functionality (% of EPs)	Functional use^a^ Mean (SD)
iEHR functionalities for LIE (*n* = 120 users)			0.97 (0.09)
I consult the laboratory results provided by the iEHR when:			
- The patient has been seen by a physician in another health establishment in Quebec;	100	99.2	
- Patients are unable to reliably report to me their recent laboratory test results or their present state of health;	100	99.2	
- The patient has no medical record in my hospital;	100	97.5	
- Patient’s laboratory test results that I require are not found in my usual information sources (e.g., the EDIS);	100	96.6	
- Caring for minor emergencies;	100	96.7	
- Caring for major emergencies (e.g., resuscitation room).	100	93.3	
EDIS functionalities for LIE (*n* = 18 users)			0.86 (0.23)
- When a patient arrives in the ED, I can verify the availability of laboratory test results directly in the EDIS.	88.0	68.0	
- I can prescribe a laboratory test directly from the EDIS.	39.1	21.7	
- I can insert and save clinical annotations when I consult a laboratory test result in the EDIS.	29.2	20.8	
LRV functionalities for LIE (*n* = 150 users)			0.83 (0.26)
The laboratory results viewer allows me:			
- To only access those patients’ test results that are produced by my hospital’s laboratory;	95.5%	91.7%	
- To access all of a patient’s laboratory test results, whether I prescribed such tests or not;	92.4	88.9	
- To generate tables and graphs for the display and analysis of lab test results;	85.0	65.4	
- To apply search criteria in order to find the lab test results that meet my needs;	80.3	63.0	
- To access patients’ test results that are produced by the laboratories in my region;	30.7	29.2	
- To electronically request a laboratory analysis and print identifying labels for the samples.	60.7	27.0	

^a^No. of functionalities used/no. of functionalities available

The next set of results pertains to the performance outcomes of LIE use in the ED, i.e., to the benefits perceived by EPs in terms of their individual efficiency and the quality of the care provided to their patients. As indicated in Table 4, there are important differences in the nature of the benefits obtained from each of the three types of systems used by EPs and in the extent to which these benefits were realized. For users of the province-wide iEHR platform, the two most important benefits were significant improvements in continuity of care and in their ability to make better clinical decisions. For EDIS users, the main benefit lies in being able to take action more quickly in an emergency situation because the patient’s test results are readily available. For LRV users, the most important benefits were the greater, quicker, and easier access to lab test results.

**Table 4:** Performance outcomes of EPs’ use of LIE systems

Performance outcomes of LIE use^a^	Mean	SD
Outcomes of iEHR use (*n* = 120 users)		
Accessing the laboratory test results provided by the iEHR:		
- Improves the continuity of my patients’ care;	4.3	0.6
- Allows me to make better clinical decisions;	4.1	0.6
- Provides me with results that I cannot obtain from my usual information sources (e.g., the EDIS);	4.0	0.8
- Improves the way in which I evaluate patients;	3.9	0.8
- Reduces duplication of the lab tests that are prescribed to my patients;	3.8	1.0
- Prevents me from missing an important result;	3.7	0.8
- Allows me to intervene more rapidly and effectively with my patients;	3.7	0.9
- Increases the safety of my patients’ care;	3.7	0.9
- Provides me with an overall view of my patients’ lab results (patients’ test history);	3.6	1.0
- Allows me to discharge patients more rapidly;	3.2	0.9
- Provides support to my clinical research or my performance measurement activities.	1.3	0.7
Overall performance outcome of iEHR use for LIE purposes^b^	3.6	0.5
Outcomes of EDIS use (*n* = 18 users)		
- I can take faster action when laboratory test results are available in the EDIS.	4.1	0.9
- The information being in one place, I gain time when I follow up on lab results through the EDIS.	3.8	1.3
- The ability to generate tables and graphs with the EDIS is very helpful in interpreting lab results.	3.6	1.3
Overall performance outcome of EDIS use for LIE purposes^d^	3.8	0.9
Outcomes of LRV use (*n* = 150 users)		
- The viewer provides most of the lab test results that I need to care for patients arriving in the ED.	4.4	0.8
- My patients’ lab test results are easier to consult in the viewer than in the paper medical record.	4.2	1.0
- It is quicker for me to access the viewer to consult patients’ previous lab test results than waiting to receive their paper medical record.	4.2	1.2
- As most of my patients reside in the region, I have little use for the iEHR because the viewer provides me with most of the lab test results that I need.	2.9	1.5
- The viewer is very useful in allowing me to access test results produced by the public laboratories in my region.	2.2	1.5
Overall performance outcome of LRV use for LIE purposes^c^	3.6	0.8

^a^As perceived by the emergency physician on Likert scales of 1 (strongly disagree) to 5 (strongly agree)

^b^Mean of the 11 performance outcomes of iEHR use

^c^Mean of the five performance outcomes of LRV use

^d^Mean of the three performance outcomes of EDIS use

Cluster analysis was used to group respondents into clusters, such that each group’s membership is homogeneous in their use of the iEHR, EDIS, and LRV systems. The SPSS Two-Step clustering algorithm was chosen, as it can handle a large number of cases, automatically determines the optimal number of clusters, and has been found to be the top-performing clustering algorithm [36]. A three-cluster solution was found to be optimal, i.e., the most interpretable and meaningful in identifying groups of EPs that could be clearly distinguished from one another. The high quality of the clusters in terms of cluster compactness and separation was confirmed by a silhouette measure [37]. As shown in Table 5, the results of the cluster analysis reveal the existence of three distinct user profiles. The 100 EPs (61%) that compose the first profile were named *iEHR-LRV-reliant* users, as they were found to make extensive use of the functionalities available in both the iEHR and an LRV system, but made little use of an EDIS. The second group of 40 emergency physicians (25%) were named *LRV-reliant* users, as they made extensive use of an LRV but limited or no use of the other two systems. Lastly, the third profile, named *iEHR-reliant* users, consists of 23 EPs (14%) who use the iEHR extensively and make some use of an EDIS but do not use an LRV.

**Table 5:** LIE user profiles

Extent of LIE use^a^	LIE user profiles			ANOVA F
	Group I *iEHR-LRV-reliant* users (*n* = 100) Mean	Group II *LRV-reliant* users (*n* = 40) Mean	Group III *iEHR-reliant* users (*n* = 23) Mean	
Use of iEHR functionalities	0.97_1_	0.01_2_	0.80_1_	557.4***
Use of LRV functionalities	0.89_1_	0.87_1_	0.03_2_	288.0***
Use of EDIS functionalities	0.09_2_	0.01_2_	0.26_1_	6.1**

Within rows, different subscripts indicate significant (*p* < 0.05) pair-wise differences between the means on Tamhane’s T2 (post hoc) test

**p < 0.01; ***p < 0.001

^a^No. of functionalities used/no. of functionalities available

In order to identify individual, organizational, and technological antecedents to LIE usage by physicians, we sought to contextualize the three user profiles that emerged from our analyses. As shown in Table 6, the three user groups do not differ significantly in terms of individual characteristics, i.e., in terms of gender, age, medical experience, or medical practice (generalist or specialist). In terms of organizational context, the *iEHR-LRV-reliant* users work in larger EDs located in urban regions, as opposed to the *iEHR-reliant* users who work in smaller EDs and the *LRV-reliant* users, more of whom practice in a peripheral region. The technological context is defined by an ED’s LIE capability, that is, by the number of functionalities made available to users within each information system used for laboratory medicine purposes. Unsurprisingly, all three user groups have access to the same LIE functionalities from the province-wide iEHR platform. However, *LRV-reliant* users perceive their LRV to include significantly more LIE functionalities than *iEHR-LRV-reliant* users, and even more than *iEHR-reliant* users who, for the most part, do not use an LRV. Moreover, *LRV-reliant* users reported that no LIE functionalities were available in their EDIS, in contrast with *iEHR-reliant* users. Thus, with the exception of the province-wide iEHR, other systems such as EDIS and LRV are not all “created equal” by software developers. Such differences in the LIE capability made available to EPs could explain why physicians differ in the extent to which they use these systems in their daily practice.

**Table 6:** Characterization of the LIE user profiles

Context and outcome of LIE use	LIE user profiles			ANOVA F
	Group I *iEHR-LRV-reliant* users (*n* = 100) Mean	Group II *LRV-reliant* users (*n* = 40) Mean	Group III *iEHR-reliant* users (*n* = 23) Mean	
Individual characteristics				
Gender [0: male, 1: female]	0.57	0.43	0.39	2.0
Age^a^	2.7	2.8	2.6	0.3
Clinical experience^b^	3.2	3.5	2.9	0.9
Medical practice [0: specialist, 1: generalist]	0.77	0.85	0.83	0.6
Organizational context				
Size of the ED^c^	2.9	2.6	2.4	3.2*
Location of the ED [0: central/urban, 1: peripheral/rural]	0.26_2_	0.70_1_	0.39	13.3***
Technological context (LIE capability)				
Number of iEHR functionalities available	6	6	6	0.0
Number of LRV functionalities available	3.7_2_	4.3_1_	1.1_3_	47.0***
Number of EDIS functionalities available	0.2	0.0_2_	0.8_1_	13.3***
Outcomes of LIE use^d^				
Performance outcome of iEHR use	3.6_1_	1.0_2_	3.2_1_	396.1***
Performance outcome of LRV use	3.5_2_	4.1_1_	1.6_3_	95.7***
Performance outcome of EDIS use	1.3	1.1_2_	2.0_1_	7.6***

Within rows, different subscripts indicate significant (*p* < 0.05) pair-wise differences between the means on Tamhane’s T2 (post hoc) test

*p < 0.05; **p < 0.01; ***p < 0.001

^a^1 = 30 years old or younger, 2 = 30–39, 3 = 40–49, 4 = 50–59, and 5 = 60 years old or older

^b^1 = 5 years or less, 2 = 5–9, 3 = 10–14, 4 = 15–19, 5 = 20–24, and 6 = 25 years or more

^c^1 = 1 EP, 2 = 2–5 EPS, 3 = 6–10 EPs, 4 = 11–20 EPs, and 5 = 21 EPs or more

^d^As perceived by the EP on Likert scales of 1 (strongly disagree) to 5 (strongly agree)

Our last set of findings pertains to differences in performance outcomes of LIE usage among the three user groups. Returning to Table 6, one finds that the first group, the *iEHR-LRV-reliant* users, receives as many benefits from their use of the province-wide iEHR system as the *iEHR-reliant* users, whereas the *LRV-reliant* users receive very limited benefits from this system. Note also that the *iEHR-reliant* EPs, the smallest group, are the only ones to benefit from their use of an EDIS, in combination with their use of the iEHR, because, in contrast to the other two groups, their EDIS includes some LIE functionalities. Recalling that the most important benefits of iEHR and EDIS use were found to be in terms of quality of care and patient safety, whereas the benefits of LRV use were more in terms of physician efficiency, it appears that the *iEHR-LRV-reliant* EPs obtain on average the highest performance outcomes for all aspects. Such differences in performance outcomes among the three groups would thus be mainly explained by the differences in the LIE capabilities, i.e., in the number of LIE functionalities available, and by the extent to which the LIEs are actually used by EPs.

## Discussion

The findings of the present study confirm that the use of a LIE in support of the emergency care process is associated with perceived improvement in performance. However, we also observed substantial variability in terms of the nature and extent of the perceived benefits. This may be attributed to the varying LIE capabilities and differences in the nature and extent of LIE usage by EPs, thus providing answers to this study’s research questions [38].

With regard to the first question, the nature of LIE usage by EPs was found to most often involve the concomitant use of two or more systems. Comparing this study to the results of prior studies cited above, a contribution lies in its identification of the polymorphous nature of LIE usage in emergency care settings. Most of the EPs in our sample appear to believe that no single system can meet all their LIE needs, not the province-wide iEHR, their hospital’s electronic medical record system, nor their department’s EDIS. Moreover, most EPs do not use their department’s EDIS for LIE purposes because these systems do not provide any LIE functionalities. These findings also provide a previously lacking empirical grounding for calls for more LIE capability to EDIS and greater interoperability of these systems with other LIE systems used by EPs [39].

As to the second research question, a cluster analysis allowed us to characterize the extent of LIE usage by EPs in a succinct and meaningful manner. The first and most prevalent user profile, named *iEHR-LRV-reliant* users, consists of EPs who make extended use of the province-wide iEHR and an LRV. This confirms that in laboratory medicine, the iEHR artifact contains information and has useful functionalities that are complementary to those found in other systems. The second group, named *LRV-reliant* users, differs from the first in that its EPs make no use of the iEHR for laboratory medicine purposes, as they appear to be satisfied with the information available in their LRV. This finding is interesting since the physicians in this group have no more LRV functionalities and no fewer iEHR functionalities available to them than physicians in the first group. But we cannot explain why these EPs make no use of the iEHR system, except for the fact that they tend to practice emergency medicine in rural rather than urban regions. A plausible explanation may be that in urban areas, patients tend to go to different hospitals or medical clinics, thus creating a need for the EPs to consult the iEHR system. In contrast, in rural areas, there is usually a single hospital and a very small number of clinics to which most of the patients go to, lowering the need for EPs to consult information contained in the provincial iEHR system.

The EPs in the third group, named *iEHR-reliant* users, differ from the other two groups in that they make, on average, very little use of an LRV. A possible explanation may be that fewer LRV functionalities for LIE, and conversely more EDIS functionalities, are available in the system used by this group. Once again, this confirms that functional design differences between LIE systems influence usage in the ED. Moreover, future research could capitalize on the notion of “extended use” to acquire a better understanding of another individual, organizational, and technological determinants of LIE usage in this context [40].

Last, it was found that the *iEHR-LRV-reliant* EPs, who make extensive use of both the iEHR and a LRV, obtain the widest range of benefits from such use in terms of efficiency, quality, and safety of emergency care. Note also that the *LRV-reliant* EPs obtain greater benefits from their use of an LRV than the first group, simply because their LRVs provide them with a greater number of LIE functionalities. In a similar fashion, *iEHR-reliant* EPs obtain greater benefits from their use of an EDIS than the second group because their EDIS artifacts provide them with more LIE functionalities. These results thus challenge system developers and vendors with regard to not only the interoperability or “openness” of the LIE software products they develop, but also concerning how to approach the functional design of these products to generate the widest range of performance benefits. Future studies could investigate the notion of “effective use” to enhance our understanding of the impacts of LIE usage on the performance of EPs [41].

## Conclusions

In this study, we sought to develop a better understanding of how EPs are using the technological tools at their disposal to consult their patients’ laboratory test results, as well as the benefits they derive from such use. Our results must be considered in light of the study’s response rate and the usual limitations associated with survey research, as there may still be biases related to the perceptual nature of the LIE performance outcome data. The variability observed in the performance outcomes by EPs could be further explained through a better understanding of the clinical environment of this use. In this regard, integrating LIE use into a process model of emergency care, such as the one proposed by the International Federation for Emergency Medicine [42], would deepen our understanding of how this use affects emergency clinical processes, and how improved performance ensues in terms of efficiency, quality, and safety. In-depth case studies of LIE usage in a variety of ED contexts should help us improve our understanding of this process and, hence, contribute to the performance of emergency physicians and departments.

The high participation rate, with respondents representing 36% of all EPs practicing in Quebec hospitals, should be interpreted as a clear sign of EPs’ interest in laboratory medicine as well as the information technologies supporting it. The great majority of our respondents indicated that they use several systems to consult their patients’ laboratory results. Even more encouraging is the fact that the more widespread this use, the higher the perceived benefits. More specifically, extensive use of the LIE allows EPs to intervene promptly with their patients and make informed clinical decisions, due in part to the laboratory results (and, in particular, abnormal and critical results) that are made available in a timely manner. In other words, extensive use of these systems allows EPs to better determine and monitor the health status of their patients, verify their diagnostic assumptions, and apply evidence-based practices in laboratory medicine (e.g., in test selection and interpretation). But in order for such benefits to be possible, it is important to ensure that EPs are provided with quality systems producing accurate, precise, up-to-date, relevant, complete, and easy-to-interpret information in a timely manner.

## Footnotes

1 As opposed to a “scale” measure that is constructed by assigning scores to patterns of responses, an “index” measure is constructed by accumulating scores assigned to individual attributes [33]. As an index combines elements not expected to be strongly intercorrelated, Cronbach’s *α* coefficient is inappropriate to test its internal validity [43].
